# Evolving Indications of Esophageal Peroral Endoscopic Myotomy (E-POEM): A Review of Expanding Applications in Esophageal Pathologies

**DOI:** 10.1007/s11894-025-01016-z

**Published:** 2025-10-10

**Authors:** Yervant Ichkhanian, John M. DeWitt

**Affiliations:** 1 Division of Gastroenterology and Hepatology, Indiana University School of Medicine, Indianapolis, IN USA; 2 Division of Gastroenterology and Hepatology, Indiana University Health Medical Center, 550 N. University Blvd, Indianapolis, UH, IN 4100, 46202 USA

## Abstract

**Purpose of Review:**

Since its introduction in 2010, peroral endoscopic myotomy (POEM) has reshaped the management of esophageal motility disorders. Originally designed for achalasia, it has expanded into a versatile therapeutic option for a range of esophageal conditions. This review summarizes current evidence on the role of esophageal POEM (E-POEM) across diverse pathologies.

**Recent Findings:**

Emerging data suggest favorable outcomes of E-POEM for non-achalasia disorders, including spastic esophageal disorders, esophagogastric junction outflow obstruction (EGJOO), diverticular disease, revisional therapy, and other rare indications. However, the evidence remains limited, and careful patient selection is essential before offering treatment.

**Summary:**

E-POEM has progressed from a procedure for classical achalasia to a promising approach for complex and refractory esophageal conditions. Its broader application may be beneficial when performed in appropriately selected patients under expert hands.

## Introduction

Peroral endoscopic myotomy (POEM) represents a significant advancement in therapeutic endoscopy, combining natural orifice transluminal endoscopic surgery (NOTES) principles with effective myotomy techniques. Since its introduction by Inoue et al. in 2010 for achalasia (1), POEM has evolved considerably in technique, patient selection, and clinical indications (2). Within the esophagus—termed esophageal POEM (E-POEM)—this approach has become especially valuable.

Achalasia, characterized by impaired lower esophageal sphincter (LES) relaxation and absent peristalsis (3), was the original and still most common indication for POEM. Over the past decade, growing operator experience, procedural refinements, and clinical demand have broadened E-POEM’s application to include other esophageal motility disorders, including spastic, post-surgical, and structural etiologies (2, 4).

In this review we list the evolving indications for E-POEM, outline its clinical development, and summarize the current evidence supporting its expanding role in non-achalasia esophageal pathologies.

## Esophageal Peroral Endoscopic Myotomy (E-POEM)

Initially developed as an endoscopic alternative to Heller myotomy (HM), POEM offers comparable efficacy with shorter postoperative hospital stays, often allowing same-day discharge in selected patients (5–7). In a landmark randomized clinical trial, clinical efficacy of POEM for achalasia at two years after intervention was noninferior to HM combined with partial fundoplication, yet gastroesophageal reflux (GER) was more common for POEM compared to HM (5). For patients with type III achalasia, POEM appears superior to HM, where an extended and tailored myotomy is often required based on pre-intervention manometry (8, 9). For example, a multicenter retrospective study of 75 patients with type III achalasia showed significantly better clinical response with POEM (98%) compared to HM (80.8%, *p* = 0.01). In this study [10], POEM permitted a longer myotomy (16 cm vs. 8 cm), a shorter procedure time (102 vs. 264 min), and a lower adverse-event rate (6% vs. 27%) (10). With increased literature and clinical experience documenting the efficacy and safety of POEM, the technique has been rapidly adopted worldwide as the first line treatment option for the management of achalasia where available (2).

## Technical Evolution of Peroral Endoscopic Myotomy Since its Inception

**Fig. 1:**
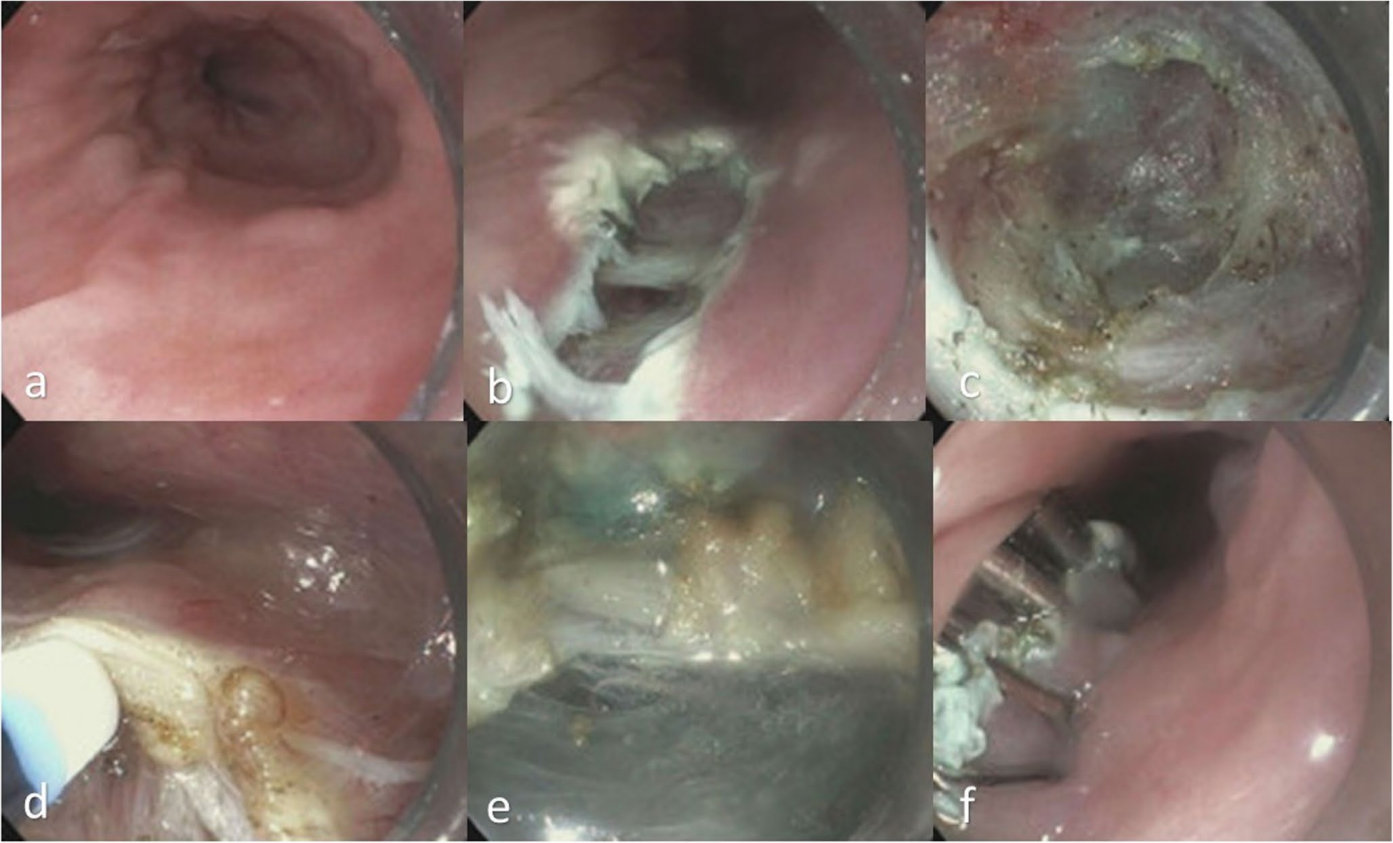
Esophageal peroral endoscopic myotomy (E-POEM) for the management of Type II achalasia. (**a**) Endoscopic view of the lower third of the esophagus indicating absence of motility and a hypertonic lower esophageal sphincter. (**z**) A longitudinal mucosectomy was performed to access the submucosal space using a hybrid knife t-type. (**c**) With the help of the endoscope clear cap the submucosal tunnel was created by injection and dissection of the submucosal fibers that extended 2 cm into the cardia. **d**-**e**) Full-thickness myotomy performed. **f**) Closure of the mucosectomy site that was created using endoscopic clips

The four steps of POEM include: mucosal incision, submucosal tunneling, myotomy, and mucosal closure (1) (Fig. 1). However, increasing recognition of post-POEM gastroesophageal reflux disease (GERD) has spurred interest in technical refinements aimed to maintain efficacy while reducing reflux risk (11). A short esophageal myotomy (< 5 cm) in type I and II achalasia achieves similar outcomes to longer myotomies (6–10 cm) with reduced procedure time (12). Limiting the gastric myotomy to 2–2.5 cm or extending myotomy only 1–2 cm beyond the LES lowers reflux and esophagitis risk while still providing normalization of esophagogastric junction (EGJ) distensibility (13). Selective circular muscle myotomy also correlates with reduced reflux symptoms and erosive esophagitis without affecting treatment success or adverse events (14). However, salvaging oblique fibers of the LES may not be required to mitigate the risk of post-procedure GERD. In a randomized trial of 115 patients with type I and II achalasia (15), oblique fiber-sparing myotomy during POEM did not reduce the incidence of reflux esophagitis or other reflux outcomes compared to conventional myotomy (15). Finally, any choice of tunnel orientation for submucosal tunnelling and myotomy are available to the endoscopist and choice may depend on patient anatomy, prior interventions, and operator preference. There appears to be a nonsignificant trend toward less reflux with anterior myotomy (16).

## Expanded Indications of E-POEM

Non-achalasia esophageal motility disorders (NAEMD) are heterogeneous and challenging conditions for clinicians to treat. Conventional therapies, including botulinum toxin injection (BTI), pneumatic dilation (PD), and Heller myotomy (LHM), have limited efficacy in these disorders (17, 18). Although randomized controlled trials are currently lacking, observational data support selective use of POEM for NAEMDs. This section explores the rationale, outcomes, and controversies for the use of POEM for NAEMD.

### Spastic Esophageal Disorders

Spastic motility disorders such as distal esophageal spasm (DES) and hypercontractile esophagus (HE) feature abnormal contractions causing dysphagia, chest pain, or regurgitation (3). DES is a rare idiopathic disorder affecting predominantly women over 60 years of age and is characterized by premature, uncoordinated distal esophageal contractions. HE is defined by HRM as ≥ 20% of swallows with a distal contractile integral (DCI) > 8000 mm Hg·s·cm and often overlaps with GERD (3).

These disorders are often refractory to pharmacologic treatments like nitrates and calcium channel blockers, which have modest efficacy and significant side effects (19). Clinical practice guidelines (2) recommend cautious use of POEM in these conditions, with use reserved for patients with severe dysphagia and weight loss after failed medical therapy. Patients with non-cardiac chest pain alone often do not benefit from esophageal myotomy (3).

POEM for spastic disorders generally requires a myotomy that is tailored to the length of the abnormal contractions on esophageal manometry and possible findings on timed barium esophagram (TBE) and endoluminal functional lumen imaging probe (EndoFLIP) (20) Therefore, myotomy of the esophageal body measuring 15–20 cm may be necessary in these patients. Since the IRP is normal in spastic disorders, myotomy of the LES is not generally required. POEM for spastic disorders is often technically challenging due to the thickened distal esophageal muscle (20).

Clinical success rates of POEM for DES and HE are consistently high. A meta-analysis of nine studies (20) involving 210 patients reported an overall pooled clinical success rate of approximately 90%, with success for DES and HE of 88% and 72%, respectively (20). In a more recent retrospective study of 106 patients, clinical success 2–3 months after POEM was 98.1% success at 2–3 months and remained 92.6% at one year (4). Preservation of the LES in patients with spastic disorders maintains clinical efficacy while potentially lowering the risk of postoperative gastroesophageal reflux (4).

### Esophagogastric Junction Outflow Obstruction (EGJOO)

EGJOO is characterized by impaired LES relaxation with preserved peristalsis. It is a heterogeneous group with primary and secondary causes (e.g., tumors, strictures, eosinophilic esophagitis, vascular compression) (3). Prevalence ranges from 3% to 21% of HRM studies, but many cases are asymptomatic or self-resolving (3). Diagnosis requires elevated integrated relaxation pressure (IRP) on HRM in both supine and upright positions, related symptoms, and supportive tests like EndoFLIP or TBE to confirm the diagnosis (21).

Recent studies have documented the role of POEM for treatment for symptomatic, primary EJOO with refractory to conservative therapy. A prospective U.S. study of 15 patients with EGJOO confirmed by HRM and EndoFLIP (22) reported a 93% clinical success at 6 months post-POEM with marked manometric and quality-of-life improvement. However, post-operative reflux was frequent, with esophagitis in 5/10 patients (22).

### Revisional or Failed Heller Myotomy

E-POEM has recently been utilized as a minimally invasive option for patients with persistent or recurrent symptoms following prior surgical or endoscopic myotomy (23). These cases may be challenging due to altered postoperative anatomy, submucosal fibrosis, and diagnostic ambiguity. Clinicians must differentiate procedural failure secondary to inadequate myotomy from recrudescence caused by other factors, such as abnormal esophageal topography or untreated GERD (24). Interpretation of HRM may be difficult in patients undergoing previous POEM or HM, and EndoFLIP or TBE can provide a more accurate assessment of EGJ distensibility and esophageal emptying respectively to guide reintervention (24).

For patients who remain symptomatic following initial POEM, a repeat POEM offers a less invasive alternative than laparoscopic surgery and a more sustainable response compared pneumatic balloon dilation and botulinum toxin injection (23). In an international cohort study examining outcomes for achalasia after initial myotomy failure, repeat POEM showed a clinical success of 76%, without a significant increase in complications compared to the initial POEM (23). The flexibility of the POEM procedure to permit the optimal choice of orientation for submucosal dissection (anterior vs. posterior) helps to avoid regions of previous intervention. In a multicenter randomized trial of 90 patients with persistent or recurrent achalasia after Heller myotomy (25), POEM achieved a significantly higher treatment success rate than pneumatic dilation (62.2% vs. 26.7%). However, POEM was associated with a nonsignificant higher (34.3% vs. 15%) rate of reflux esophagitis compared with PD (25). Treatment choice should be individualized, factoring in prior surgery, reflux risk, anatomical feasibility, patient preference, and local expertise.

### Sigmoid Esophagus/End-Stage Achalasia

End-stage or sigmoid-type achalasia is marked by a massively dilated, tortuous esophagus with absent peristalsis and poor clearance, resulting in severe dysphagia, regurgitation, and malnutrition. In these cases, POEM may serve as a palliative option by disrupting the non-relaxing LES to improve bolus transit. The procedure poses technical challenges, including mucosal and submucosal fibrosis, distorted anatomy, difficult orientation, and loss of tissue planes (26). Despite this, expert centers report safe, effective completion in selected patients using advanced techniques like fluoroscopic guidance and modified tunnelling. POEM in this context is best viewed as symptom-directed palliation rather than definitive therapy (27).

A meta-analysis of 11 studies encompassing 428 patients with sigmoid or megaesophagus found pooled technical success of 98.3% and clinical success of 89.4% (sigmoid: 87.9%, megaesophagus: 88.4%). Adverse event rates ranged from 0 to 47%, but most principally mild in severity. Serious events reported in only a few individual series (28).

### Zenker’s and Non-Zenker’s Diverticulum (Z-POEM/D-POEM)

**Fig. 2:**
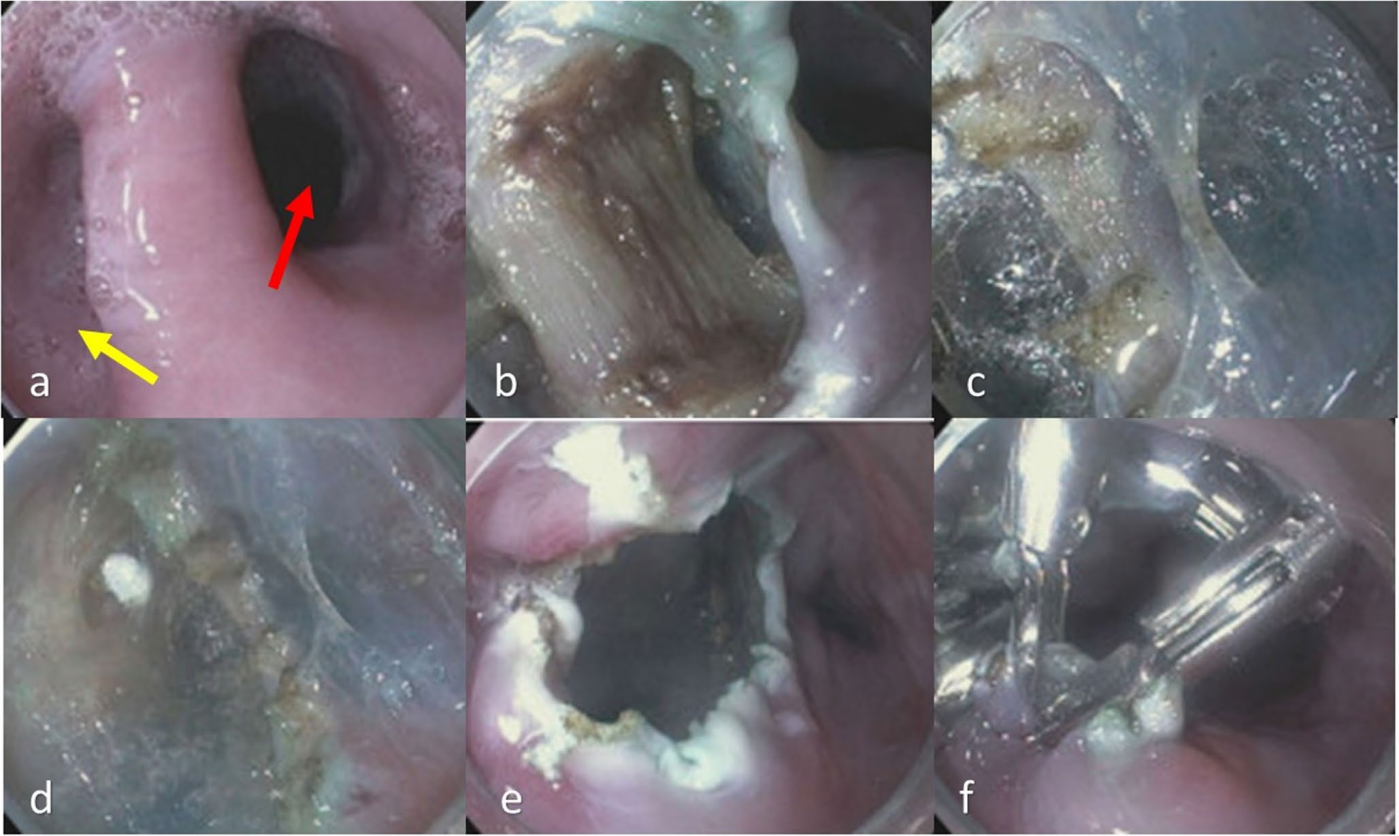
Zenker peroral endoscopic myotomy (Z-POEM) for the management of a moderate size Zenker’s diverticulum. (**a**) Endoscopic view showing the esophageal lumen (red arrow) and the pouch of the Zenker’s diverticulum (yellow arrow). (**b**) The submucosa overlying the septum exposed post submucosal injection and longitudinal mucosal incision using a hybrid T-knife. (**c**) Submucosal tunneling performed with further injection and dissection on the pouch side and the esophageal wall further exposing the cricopharyngeal muscle. (**d**) Myotomy was then performed on the septum to the base of the pouch and 1–2 cm distal onto the muscularis propria of the esophageal wall. **e**-**f**) The submucosal entry site closed with multiple clips

The submucosal tunnelling technique of POEM has been adapted to treat esophageal diverticula, particularly Zenker’s diverticulum (29) (Fig. 2). Z-POEM involves mucosal incision, submucosal tunnelling to the septum, and endoscopic myotomy of the cricopharyngeal muscle/septum under direct visualization within the tunnel (30). This technique offers advantages over flexible endoscopic septotomy (FES), which entails septum division without submucosal tunnelling. Compared to FES, Z-POEM appears to have a lower recurrence of symptoms and minimized perforation risk (30). A meta-analysis of 11 studies involving approximately 357 patients demonstrated that Z-POEM is highly effective, with a technical success rate of 96.3% and clinical success of 93.0%. The procedure was associated with an adverse event rate of 12.4% and a recurrence of symptoms in 11.2%. Compared to FES, Z-POEM achieves significantly higher clinical success while maintaining comparable recurrence outcomes with better safety profile(29, 31).

The role of POEM was further expanded for the management of epiphrenic or mid-esophageal diverticula (D-POEM). Technically, D-POEM usually combines myotomy of both the septum of the diverticulum any concurrent esophageal motility disorder (32). The technical and clinical success for the D-POEM is reported to be similar to Z-POEM at 95–100% and 86–97%, respectively (33).

## Current Controversies and Limitations

I. Limited High-Level Evidence

- Most data on E-POEM in non-achalasia disorders derive from observational studies and small case series, with few randomized controlled trials (table1). Patient heterogeneity and variable outcome definitions challenge generalizability.

**Table 1:** Level of evidence per peroral endoscopic myotomy (POEM) indication

Indication	Typical Outcome	Evidence Level	
Achalasia (Types I-III)	High efficacy, long-term durability	High (RCTs, meta-analyses)	
Distal Esophageal Spasm (DES)	Improved symptoms and manometry	Moderate (case series, cohort)	
Jackhammer Esophagus	Significant symptom relief	Moderate (small studies)	
EGJ Outflow Obstruction (EGJOO)	Variable, depends on underlying cause	Low to moderate (select studies)	
Failed Heller Myotomy	Effective as revisional therapy	Moderate (multicenter experience)	
Sigmoid Esophagus	Palliative improvement, feasible	Low (observational, expert opinion)	
Zenker’s Diverticulum (Z-POEM)	Reduced recurrence vs. stapling	Moderate (early studies, good results)	
Scleroderma-associated Dysfunction	Limited data, case-based support	Low (case reports, pilot studies)

II. Risk of Post-POEM GERD and its Management

- Post-POEM GERD remains a clinical concern and is the most common long-term complication of the procedure (5). Silent reflux is common (34, 35) and rare complications such as de novo adenocarcinoma (36) have been reported. Routine proton pump inhibitor (PPI) therapy and endoscopic or manometric follow-up are advised (2). In a retrospective review of 252 POEM patients from 2011 to 2022, 52% developed reflux esophagitis, and 12% had severe (LA grade C/D) disease (11). Emerging anti-reflux strategies including simultaneous POEM with transoral incisionless fundoplication (TIF), simultaneous POEM with endoscopic fundoplication (POEM-F) and post-procedure TIF and laparoscopic cruroplasty (cTIF) with are still considered investigational (37–39).

III. Patient Selection

- Proper selection of patients for POEM to treat non-achalasia esophageal motility disorders is critical as differentiating functional from structural causes of dysmotility may avoid overtreatment. Comprehensive pre-procedure workup including clinical history, HRM, endoscopy, barium studies, and EndoFLIP or imaging is essential. Clinical decision-making is nuanced, particularly for mild or fluctuating symptoms.

IV. Impact of Daily Opioid Use

- Chronic opioid exposure can cause acute and chronic esophageal dysmotility resulting in an elevated LES pressure, impaired LES relaxation, and spastic or obstructive manometric patterns. These opioid-related changes can mimic primary motility disorders. Temporary cessation or tapering of opioids may not relieve symptoms in these patients. Repeat physiology testing off medications may clarify the role of opioids and prevent unnecessary procedures like E-POEM (40). Furthermore, in a propensity score–matched study (41), POEM achieved a significantly lower clinical response rate in opioid users compared to nonusers (79.7% vs 93.8%).

## Future Directions

As E-POEM continues to evolve, several promising innovations and research priorities are emerging. Diagnostic integration of functional lumen imaging probe (FLIP) manometry offers real-time assessment of EGJ distensibility and contractility, aiding both diagnosis and intraoperative decision-making. Artificial intelligence-assisted interpretation of HRM and endoscopic images may enhance diagnostic accuracy, reduce interobserver variability, and support standardized treatment planning. Lastly, clinical trials and registries are crucial to identify which patients with non-achalasia disorders may benefit most from POEM.

## Conclusion

E-POEM has rapidly evolved from a novel intervention for achalasia into a versatile, minimally invasive treatment for a range of esophageal motility and structural disorders. Its success lies in its adaptability, safety profile, and potential to address conditions previously resistant to medical or surgical therapy.

However, as the field matures, the emphasis must now shift toward evidence generation, technique optimization, and multidisciplinary collaboration. With ongoing technological innovation, improved diagnostic tools, and a growing body of clinical experience, E-POEM is well positioned to become a cornerstone of modern esophageal therapy—particularly when patient selection, procedural planning, and post-procedure care are guided by best practices and evolving data.

## Key References

- Tatsuta T, Inoue H, Shimamura Y, Iwasaki M, Ushikubo K, Yamamoto K, et al. Peroral endoscopic myotomy in spastic esophageal disorders: Clinical outcomes and optimal approaches. Dig Endosc. 2025;37 (7):758 − 65.

- Great summary on the role of Peroral endoscopic myotomy (POEM) in treating spastic esophageal disorders, including type III achalasia, distal esophageal spasm, and jackhammer esophagus, with durable outcomes at one year. Authors pointed out that Lower Esophageal Sphincter (LES) preserving POEM for distal esophageal spasm and jackhammer esophagus achieved similar efficacy to gastric myotomy while showing a trend toward reduced gastroesophageal reflux.

- Wessels EM, Masclee GMC, Bastiaansen BAJ, Fockens P, Bredenoord AJ. Incidence and risk factors of reflux esophagitis after peroral endoscopic myotomy. Neurogastroenterol Motil. 2024;36(6):e14794.

- The authors re-emphesized the importance of diagnosing and managing post POEM reflux. They reported that about half of patients developed reflux esophagitis within one year after peroral endoscopic myotomy, with 12% experiencing severe disease. They showed that longer full-thickness myotomy, higher alcohol use, and overweight increased risk, while prior myotomy or pneumatic dilation reduced risk, highlighting the importance of predictive factors for patient selection and prevention.

- Ichkhanian Y, Brewer Gutierrez O, Roman S, Yoo IK, Canakis A, Pawa R, et al. Role of functional luminal imaging probe in the management of postmyotomy clinical failure. Gastrointestinal Endoscopy. 2022;96 (1):9–17.e3.

- In this study, the diagnostic challenge post failed myotomy in patients with achalasia was highlted. They suggested the role of functional luminal imaging probe (FLIP) as an aid in refining diagnosis and guiding management in over half of cases with abnormal findings. Patients with both abnormal integrated relaxation pressure and distensibility index on FLIP had the highest success following LES-directed retreatment, highlighting FLIP’s complementary role alongside HRM in post-myotomy evaluation.

## Data Availability

No datasets were generated or analysed during the current study.

## Abbreviations

Peroral endoscopic myotomy: POEM
Lower esophageal sphincter: LES
Natural orifice transluminal endoscopic surgery: NOTES
Heller myotomy: HM
Gastroesophageal reflux: GER
Esophagogastric junction: EGJ
Non-achalasia esophageal motility disorders: NAEMD
Botulinum toxin injection: BTI
Pneumatic dilation: PD
Distal esophageal spasm: DES
Distal contractile integral: DCI
Timed barium esophagram: TBE
Endoluminal functional lumen imaging probe: EndoFLIP
Esophagogastric junction outflow obstruction: EGJO
POEM + endoscopic fundoplication: POEM-F
Transoral incisionless fundoplication and laparoscopic cruroplasty: cTIF
